# ^13^C, ^27^Al and ^29^Si NMR Investigation of the Hydration Kinetics of Portland-Limestone Cement Pastes Containing CH_3_-COO^−^-R^+^ (R=H or Na) Additives

**DOI:** 10.3390/ma15062004

**Published:** 2022-03-08

**Authors:** Anton Mazur, Peter Tolstoy, Konstantinos Sotiriadis

**Affiliations:** 1 Physical Chemistry Department, Institute of Chemistry, Saint Petersburg State University, Universitetsky pr. 26, 198504 Saint Petersburg, Russia; peter.tolstoy@spbu.ru; 2 Department of Theoretical and Applied Mechanics, Institute of Theoretical and Applied Mechanics of the Czech Academy of Sciences, Prosecká 809/76, 19000 Prague, Czech Republic; sotiriadis@itam.cas.cz; 3 Department of Building Materials and Products, South Ural State University, pr. Lenina 76, 454080 Chelyabinsk, Russia

**Keywords:** Portland-limestone cement, organic additives, hydration kinetics, NMR spectroscopy

## Abstract

The hydration kinetics of Portland-limestone cement pastes with organic additives in the form of acetic acid and sodium acetate were studied by using solid-state ^13^C, ^27^Al and ^29^Si NMR spectroscopy. The evolution of the relative content of various phases was monitored over the period of one month: amorphous and crystalline calcite (in ^13^C spectra), ettringite, aluminum in C-S-H gel, calcium aluminates and calcium hydroaluminates (in ^27^Al spectra), as well as alite, belite and silicon in C-S-H gel (in ^29^Si spectra). The retarding effect of the additives on cement hydration at early age was demonstrated. We show that the kinetics of phase assemblage formation is influenced by the acetate ion adsorption on the surface of the anhydrous cement components and hydrated phases. The kinetics of formation of ettringite in the cement paste, depending on the addition of acetic and or sodium acetate, is discussed in the context of potential thaumasite sulfate attack.

## 1. Introduction

Cement-based building materials are among the most used in modern construction. Despite their widespread application and the large amount of information regarding the chemistry of cement pastes [1,2], several issues regarding their properties during the hydration process and the effect of various environmental factors on the hardened material are still not clear. One of these factors is the chemical sulfate attack, which might occur in the form of conventional (ettringite or gypsum formation) or thaumasite sulfate attack (TSA) [3,4]. The former process is associated with the active formation and growth of expansive ettringite and gypsum crystals in the cement paste matrix, while the latter involves the formation of thaumasite microcrystals, occurring readily in the presence of carbonate ions and at temperatures close to the freezing point of water, about 0–5 °C. The amount and volume occupied by these minerals increase during the attack, developing internal stresses that induce cracks in the hardened cement paste. Moreover, as more silicon is available for the formation of thaumasite during TSA, which is derived from the continuously deteriorating calcium silicate hydrate phase, cement paste gradually transforms into a non-cohesive mass, further contributing to the destruction of building structures. TSA is particularly dangerous for Portland-limestone cement materials, because the presence of calcium carbonate facilitates this type of chemical attack.

To reduce the effect of deleterious external factors and optimize the hydration process and the properties of the obtained cement pastes, various additives based on mineral, organic or multicomponent substances are actively used [5,6,7,8]. However, although complex multicomponent additives are widely used, the hydration process in the presence of simple organic substances has not been fully investigated. In this work, we decided to study the effect of commonly used additives, such as sodium acetate and its parent acid, on the hydration kinetics of Portland-limestone cement. Sodium acetate is used as anti-freeze additive [9], as well as for reducing the permeability of concrete to water and sulfate ions [10]. Acetic acid, in turn, is used to control the hardening time of cement [11]. Moreover, an amount of additive about 3% by cement mass was observed as optimal for achieving the maximum strength of the cement stone.

^13^C, ^27^Al and ^29^Si NMR spectroscopy was selected as the main research tool. The technique allows for obtaining information about the local environment of the investigated nuclei [12]. The advantage of the applied method, in comparison with X-ray diffraction research methods, is that NMR spectroscopy allows for recording signals from both the amorphous and crystalline parts of the investigated samples. In hydrated cements, the amorphous part mainly consists of the calcium silicate hydrate phase, which provides most of the strength of the hardened cement paste.

The main aim of this work was to study the kinetics of the phase assemblage formation in Portland-limestone cement pastes in the presence of acetic acid and its sodium salt, as well as to elucidate the mechanism behind the observed hardening rate and appraise the changes of its strength properties, based on the data obtained. Research on cement hydration that assesses a large number of nuclei with NMR spectroscopy is scarce in the literature. The applied method is supposed to provide detailed structural and quantitative information about the occurring phase changes.

## 2. Materials and Methods

Cement pastes were prepared with a type CEM II/A-L 42.5N Portland-limestone cement (SLK Cement–Sukhoy Log, Sverdlovsk Region, Russia), distilled water and p.a. organic additives (acetic acid and sodium acetate). A water-to-cement ratio of 0.45 was used. Organic additives amounted to 3% by cement mass each, both added to the mixing water. Cement pastes were cast in cylindrical plastic molds (12 mm in diameter; 30 mm in height), where they remained sealed for 24 h. After demolding, the specimens were immersed in distilled water and kept throughout the investigated hydration periods. To study the kinetics of the changes in the phase composition of the pastes at certain ages (after 1, 2, 3, 5, 7, 14 and 34 days), about 150 mg of hardened material was scraped off the specimens’ end and placed in paper bags to prevent any further hydration. Such quantity was sufficient for conducting the NMR experiment. In this work, the following nomenclature was used to label the samples: **C** (cement paste without additives), **CAA** (cement paste with acetic acid) and **CSA** (cement paste with sodium acetate). The stage of hydration was indicated by the age of the cement paste, which was added to the sample marker.

Prior to hydration, the mineralogical composition of the employed cement was determined with X-ray powder diffraction (XRPD) analysis; the results are summarized in Table 1. XRPD analysis was performed at room temperature, using CuKα radiation 2-theta range 5–80°, step 0.0203°, voltage 30 kV and current 10 mA. Qualitative X-ray phase analysis was carried out with the software PDXL 2.8.4.0 (Rigaku, Tokyo, Japan), with connection of PDF-2 database (International Diffraction Data Center, ICDD). Quantitative phase analysis (wt.%) was carried out by applying the Rietveld method [13] on the obtained full-profile data with the software TOPAS 4.2 (Bruker, Billerica, MA, USA).

NMR experiments were performed by using an Avance III 400 WB spectrometer (Bruker, Billerica, MA, USA) at constant magnetic field of 9.4 T. ^13^C, ^27^Al and ^29^Si nuclei were studied and characterized by the respective resonance frequencies of 100, 104 and 86 MHz. A probe that is able to rotate the samples at the magic angle to the direction of the constant magnetic field (stabilization accuracy of the rotation frequency ±4 Hz) and stabilize their temperature (temperature stabilization accuracy ±1 °C) was used. Powder samples were loaded on a 4 mm zircon oxide rotor and rotated at a frequency of 12.5 KHz at 20 °C. Tetramethylsilane, for ^13^C and ^29^Si nuclei, and 1 M∙D_2_O AlCl_3_ solution, for ^27^Al nuclei, were used as external references.

All the spectra were recorded by using a single-pulse sequence. The duration of the exciting impulses was 2.5, 4.5 and 2.5 μs; the relaxation delay was 4, 2 and 4 s; the number of scans was 1024, 512 and 1024 for ^13^C, ^27^Al and ^29^Si nuclei, respectively.

Deconvolution of spectra into Gaussian-shape individual components was performed by the least squares method, using the software Origin 9.0. (OriginLab Corporation, Northampton, MA, USA) For all the spectra, the results of approximation were obtained with a coefficient of determination *R^2^* higher than 0.8. Since single-pulse sequence was used to record the NMR spectra, the relative integrated intensities of the signals can be interpreted as mole fractions of the corresponding phase components.

## 3. Results

### 3.1. ^13^C

**Figure 1:**
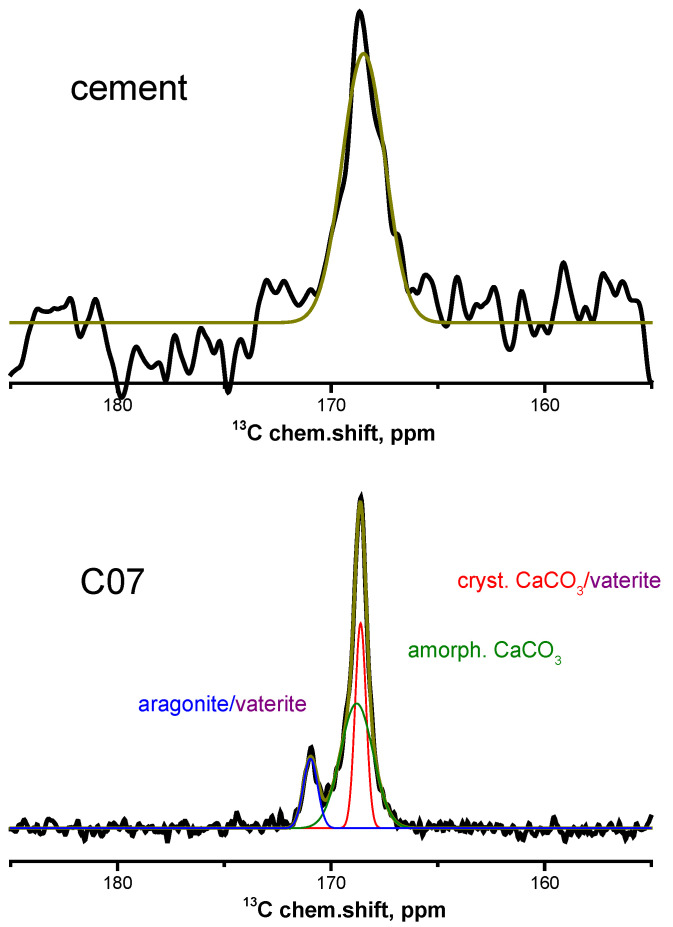
^13^C NMR spectra of the (**top**) anhydrated Portland-limestone cement, and (**bottom**) cement paste sample without additives on the 7th day of hydration.

The ^13^C NMR spectrum of the anhydrated cement (Figure 1 (top)) shows a single broad asymmetric line at a chemical shift of about 168.7 ppm. Most likely, this line originates from amorphous calcium carbonate and, possibly, small amounts of calcite and dolomite, which are contained in cement, according to the phase composition obtained from the XRPD analysis (Table 1). An effort to perform component deconvolution in this spectrum was not attempted, because its line shape is quite broad and the ^13^C chemical shifts of the carbonate compounds fall close to each other [14]. The ^13^C NMR spectra obtained at different ages of cement hydration show two (except for **CAA** and **CSA** at 1 d) clearly distinguished peaks (Figure 1 (bottom) is an example of the ^13^C NMR spectrum of the sample C07; the other spectra are provided in Supplementary Figures S1a–S3a). Moreover, the peak at about 168.5 ppm consists of unresolved narrow and broad signals; the deconvolution into two peaks is justified in Supplementary Figure S1c,d. We note, however, that the precision of the deconvolution of unresolved signals might suffer from larger errors and a certain degree of caution should be exercised when analyzing these results. The position of the signal at about 171 ppm remains virtually unchanged over time. However, the narrow component of the signal at 168.5 ppm was slightly shifted to the weak field at the initial stage of hydration, and the broad component was shifted to the strong field throughout the entire hydration period, with the exception of the **CSA** sample, for which this component was shifted, on the contrary, to the weak field. The signals mentioned are contributed also by the presence of calcium monocarboaluminate hydrate, forming during the hydration process. The time dependences of the ^13^C chemical shift values are shown in Supplementary Figures S1b–S3b in Supporting Information.

According to the literature, all the observed signals correspond to CO_3_^2^^−^ structural units of various amorphous and crystalline modifications of calcium carbonate and dolomite [14,15,16]: the broad signal at about 168.5 ppm corresponds to amorphous calcium carbonate and to metastable ikaite, which is possibly contained in the samples; the narrow signal corresponds to calcite, dolomite and vaterite; and the signal at about 171 ppm corresponds to carbon atoms in aragonite and vaterite. According to Reference [17], the two narrow signals from carbon nuclei in the structure of vaterite correspond to its two most probable crystal structures.

### 3.2. ^27^Al

**Figure 2:**
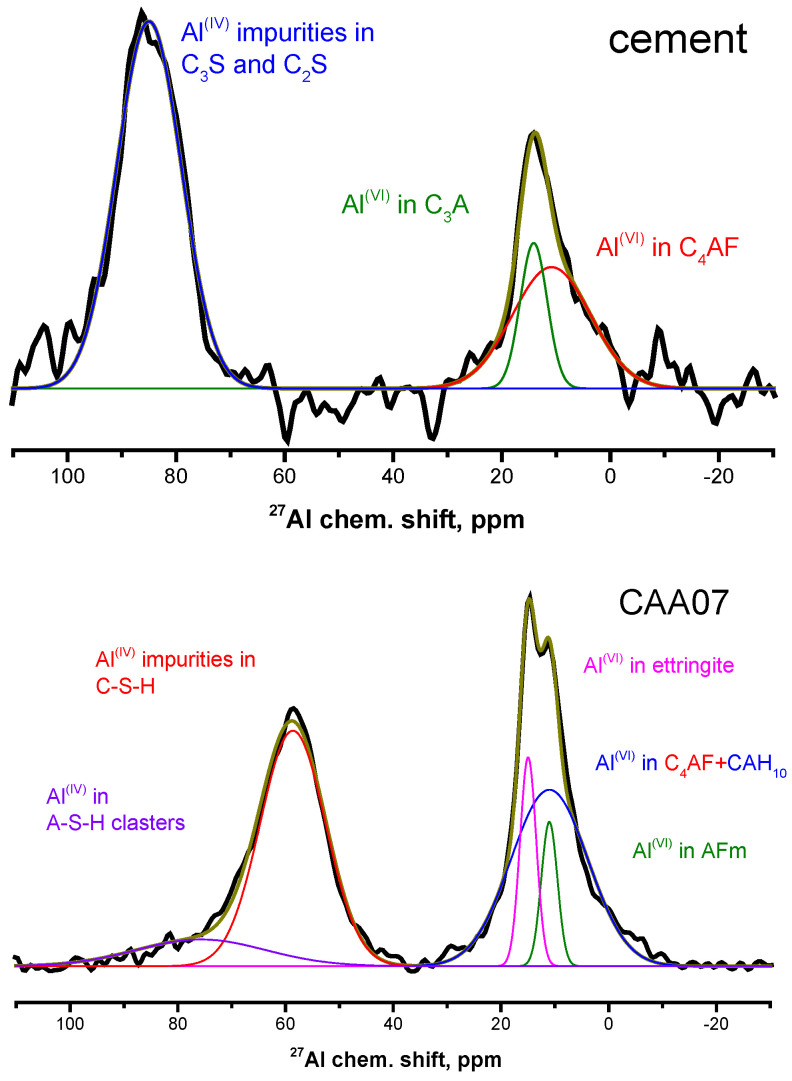
^27^Al NMR spectra of the (**top**) anhydrated Portland-limestone cement, and (**bottom**) cement paste sample with acetic acid addition at the 7th day of hydration.

The ^27^Al NMR spectrum of the anhydrated cement (Figure 2 (top)) shows two isotropic signals. The signal at 85 ppm corresponds to aluminum atoms in tetrahedral environment of oxygen atoms Al^(IV)^, which are in the form of impurities in alite and belite [18]. The signal at about 10 ppm corresponds to aluminum atoms in octahedral environment of oxygen atoms Al^(VI)^. This signal consists of two spectral components: the narrow component at about 15 ppm corresponds to aluminum atoms in Ca_3_Al_2_O_6_ (C_3_A), and the broad one at about 10 ppm to C_4_AF [18].

In the ^27^Al NMR spectra of all studied hydrated samples (Figure 2 (bottom) shows the ^27^Al NMR spectrum from the **CAA07** sample; the rest of the spectra are shown in Supplementary Figures S4a–S6a), it can be seen that the signal from the aluminum atoms Al^(IV)^ in anyhydrated alite and belite practically disappears already on the first day of hydration; there is only a weak signal at this age for the **CAA** and **CSA** samples. At the same time, a broad asymmetric signal appears at about 65 ppm that can be described as the sum of the two components at 60 and 75 ppm. Both correspond to aluminum atoms Al^(IV)^ in the amorphous cement hydration gel. In fact, the line at about 75 ppm is typical for aluminum incorporation in the C–S–H gel, while the line at around 60 ppm for aluminum atoms in an unstable aluminum silicate hydrate (A–S–H) gel, forming near the surface of clinker grains at conditions of calcium shortage and excess aluminum [19]. In this case, the total relative integrated intensity of the signals in the given spectral region decreased with increasing the hydration time.

For the **C** and **CSA** samples, a broad asymmetric signal in the region of Al^(VI)^ chemical shifts can be described also by two components: a narrow one at 15 ppm and a broad component, whose chemical shift decreased from 14 to 10.5 ppm from the 1st to the 7th day, and then increased to 14.5 ppm by the end of the investigated time interval. For the **CAA** sample, there is another narrow component, in this region of the spectrum, at about 11 ppm (see the example in Figure 2, bottom). The narrow intense line at about 15 ppm corresponds to aluminum atoms in ettringite [12]. The narrow line of weak intensity at about 11 ppm corresponds to calcium monocarboaluminate hydrate (AFm) [19]. The broad line, whose position varies in the range of about 10.5–14.5 ppm, corresponds to signals from several aluminum hydrates and calcium hydroaluminates of different compositions. According to the behavior of the chemical shift of the broad line under consideration, it can be assumed that redistribution of coexisting phases occurs, from aluminate hydrate (AH_3_) through mono-(CAH_10_) and dicalcium hydroaluminate (C_2_AH_8_) to tricalcium hydroaluminate (C_3_AH_6_) [12].

### 3.3. ^29^Si

**Figure 3:**
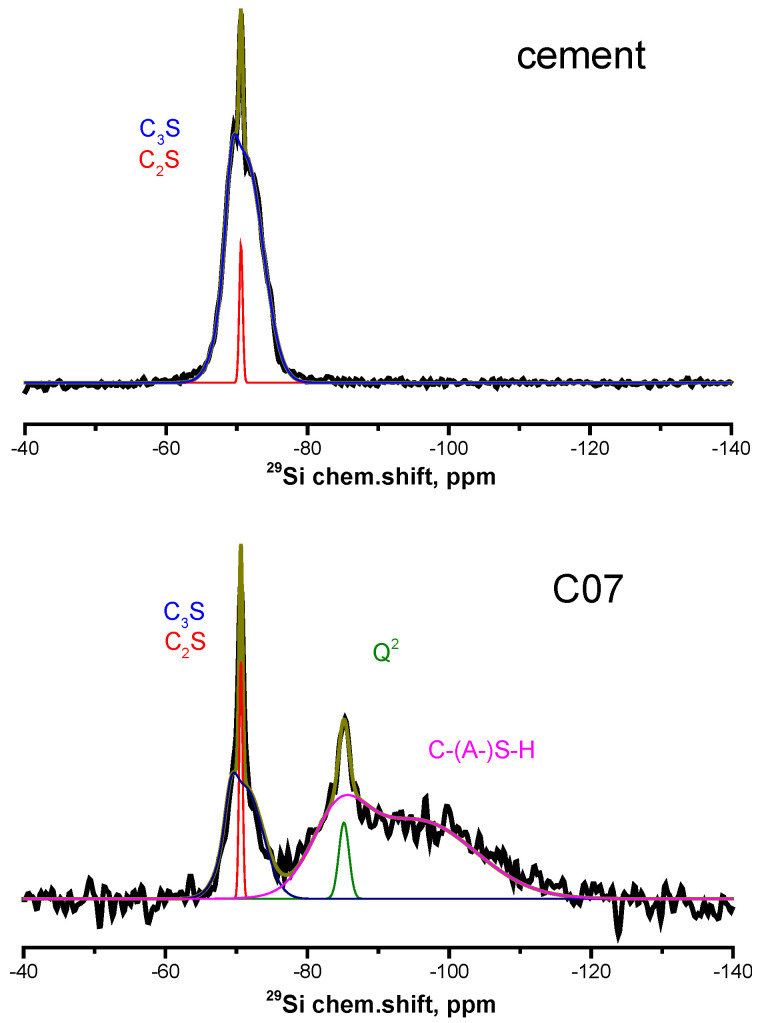
^29^Si NMR spectra of the (**top**) anhydrated Portland-limestone cement, and (**bottom**) cement paste sample without additives at the 7th day of hydration.

The ^29^Si NMR spectrum of the anhydrated cement (Figure 3 (top)) shows a single signal, which is superposition of a narrow line at about −70.6 ppm from silicon atoms in the structure of belite and a broad asymmetric line in the range from −65 to −75 ppm, which corresponds to silicon atoms in the structure of alite [20] that are located in various local environments.

During the initial stage of hydration of all three cement mixtures, broad unresolved lines appear in the spectrum initially in the range from −75 to −95 ppm, and then the specified range expands to chemical shift values of about −120 ppm (Figure 3 (bottom)) shows the ^29^Si NMR spectrum from the sample **C07**; the rest of the spectra are shown in Supplementary Figures S7–S9). This behavior corresponds to the appearance of an inhomogeneous phase, containing silicon atoms in tetrahedral environment of oxygen atoms, which are characterized by the presence of one to two linked silicon tetrahedra (Q^1^ and Q^2^ structural elements, from −75 to −90 ppm) or of three to four linked silicon tetrahedra (Q^3^ and Q^4^ structural elements, from −90 to −120 ppm) [12]. The presence of the former two structural elements in the cement paste characterizes the formation of the mostly amorphous C–(A−)S−H gel, while the latter two point to the formation of crosslinked silicate chains (C−(A−S−H of low Ca/Si ratio)) and amorphous hydrous silica [21].

At the later stages of hydration, a narrow line at about −86 ppm appeared in the spectra. This line corresponds to silicon atoms in Q^2^ structural elements of the cement paste, in paired (Q^2^_P_) and/or bridged (Q^2^_b_)silicon tetrahedra in silicate chains, which form the bulk structure of the resulting cement paste [22].

## 4. Discussion

**Figure 4:**
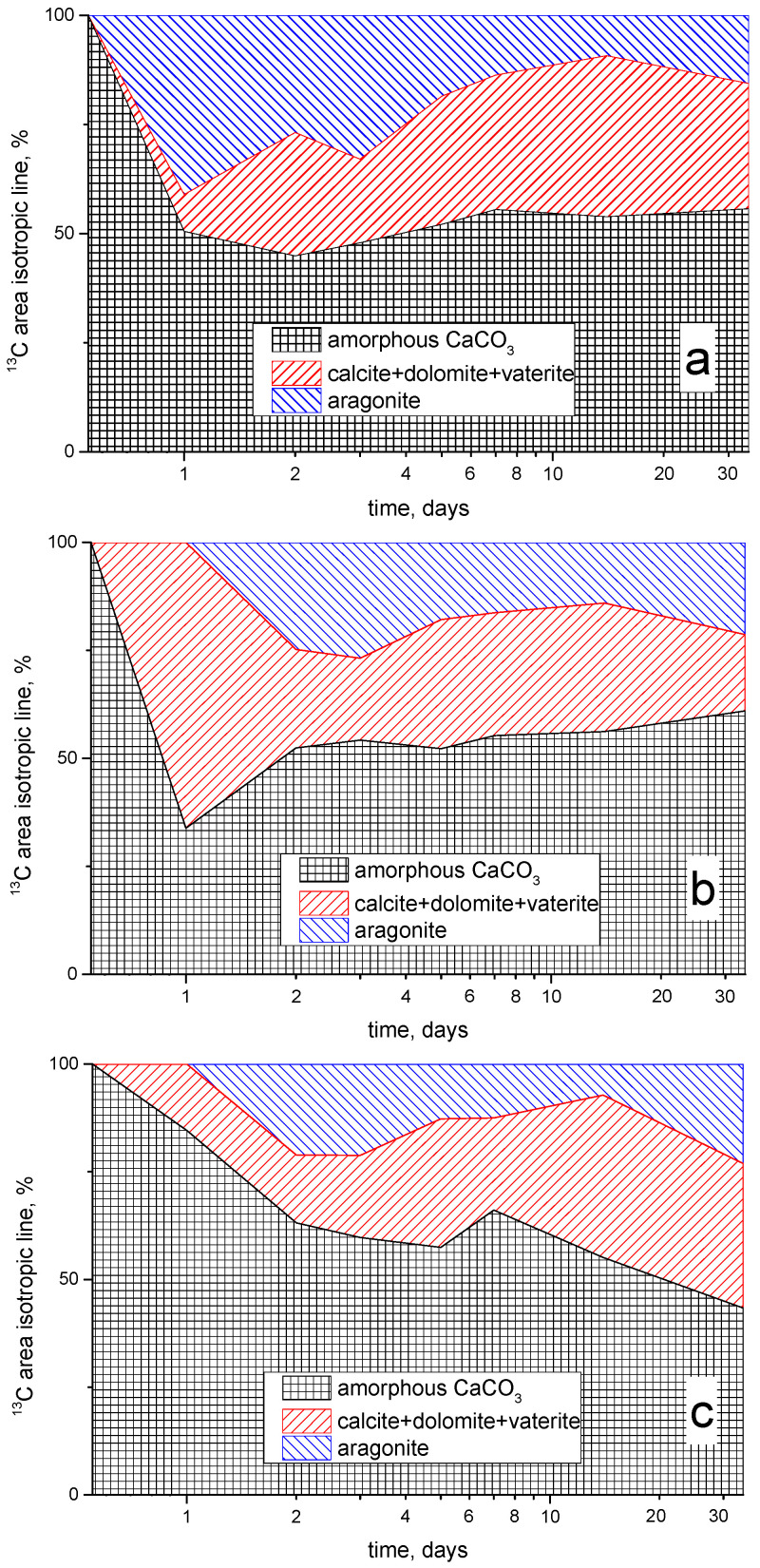
Time-evolution (in logarithmic scale) of the relative integrated areas of the isotropic components recognized in the ^13^C NMR spectra of the samples: **C** (**a**), **CAA** (**b**) and **CSA** (**c**).

Figure 4 shows the time dependences of the relative integrated intensities of the ^13^C NMR signals for all three studied samples. It can be seen that the intensities of the lines corresponding to amorphous calcium carbonate sharply decrease at the initial stage of the hydration process for **C** and **CAA** samples, and then a slight increase is observed. For the **CSA** sample, a gradual decrease in the relative proportion of amorphous calcium carbonate is observed. The proportion of calcite increases for all the samples, while that of aragonite decreases, as is especially noticeable for **C** sample. However, at the end of the studied hydration period (15–34 days), the relative content of calcite and aragonite essentially stabilizes and for **CAA** sample even slightly reverses. It should be noted that a significant amount of aragonite is observed on the first day of hydration only for **C** sample.

The increase in the content of the poorly soluble calcium carbonate polymorphs (calcite and aragonite), as noted for all samples, leads to their precipitation in the pores of the hardening cement paste, and this might cause an increase in its strength and a decrease in porosity [1,2]. The smallest amount of these calcium carbonate polymorphs is observed for the sample **CAA**.

Evidently, the formation of aragonite in the samples containing organic additives begins only on the second day of hydration, while the fraction of the initial amorphous CaCO_3_ in the **CSA** sample, on the first day, is much lower than for the other samples. This observation can be attributed to the fact that the acetate ion (CH_3_COO^−^) can be adsorbed on the surface of the anhydrated cement microparticles and prevent their hydration [10,23], and also the crystallization of new phases [24]. However, in the case of acetic acid addition, the acidity of the pore solution increases, and this increase, at the initial stage of hydration, contributes to the dissolution of the fine particles of the anhydrated cement. At the same time, the presence of sodium cations hinders this process, forming a weakly alkaline medium in **CSA** mixture.

It is worth noting that it is not possible to quantify the amount of calcium monocarboaluminate hydrate (AFm) in **CAA** sample from ^13^C NMR spectra, although this compound is resolved in the corresponding ^27^Al NMR spectra. This is because of the negligible ^13^C NMR chemical shift difference between AFm phase and other calcium carbonates (calcite and vaterite) that prevents a reliable deconvolution of the overlapped signals [14].

**Figure 5:**
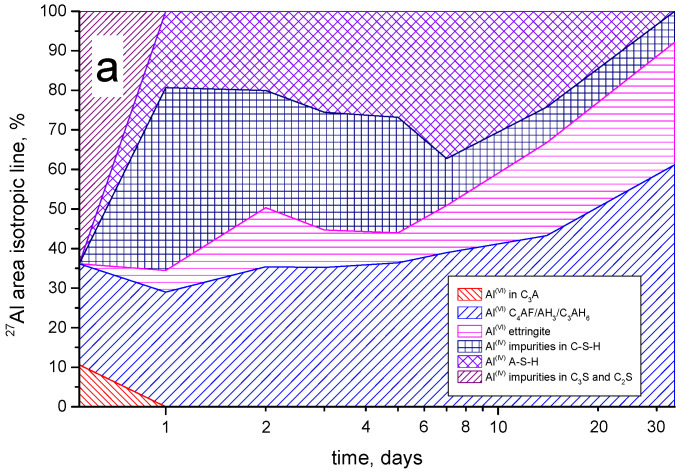

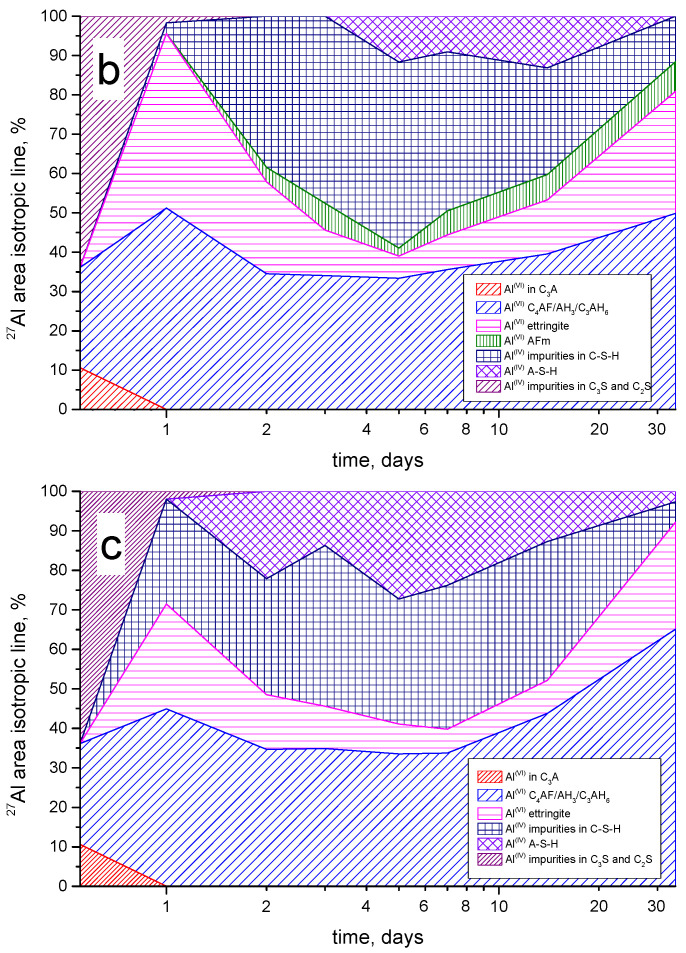
Time-evolution (in logarithmic scale) of the relative integrated areas of the isotropic components recognized in the ^27^Al NMR spectra of the samples: **C** (**a**), **CAA** (**b**) and **CSA** (**c**).

Figure 5 illustrates the time dependences of the relative integrated intensities of the ^27^Al NMR signals for all three studied samples. Considering the observed changes in the intensities of the aforementioned spectral components, it can be concluded that, after the initial dissolution of the aluminate phases of the cement used, a large amount of aluminate hydrate forms, whose quantity gradually decreases. Then the amount of various compounds in the form of C–S–H, A–S–H or C–(A–)S–H gels grows in volume, further gradually decreasing. Finally, a gradual increase in the amount of various calcium hydroaluminates and ettringite is observed.

It should be noted that, for the **CAA** and **CSA** samples, on the first day, there is an insignificant amount of residual aluminum impurity in C_2_S and C_3_S. This can be associated with incomplete hydration of the anhydrous cement particles, due to the adsorption of the acetate ions on their surface.

The diagrams of Figure 5 show that, for all samples, the amount of ettringite initially increased and then decreased, and, at later stages, it again increased [2]. According to the generally accepted theory of hydration of aluminum-containing cements, ettringite crystallizes in two stages. In the initial stage, long narrow crystals form, which contribute to the initial binding of the hydrated cement grains. Later, during the deceleration of the hydration process, the initially formed ettringite recrystallizes in the form of large crystals in the voids of the matrix. Moreover, for the **C** sample, recrystallization of ettringite practically did not occur, while, in the **CAA** sample, the amount of the primary and secondary ettringite is noticeably larger than for all the other samples. The presence of acetate groups in the **CAA** and **CSA** samples can partially replace the sulfate groups [24]. Hence, the excess of the latter facilitates the formation of primary ettringite, whose content is larger than that in the **C** sample, resulting in the kinetics observed.

Since the surface of the aluminum-containing clinker phases is more electronegative than that of C_3_S and C_2_S [11], their dissolution occurs faster and, in parallel, a deficiency of calcium arises. Thus, in the **CAA** and **CSA** samples, at the early stages of hydration, an increased amount of aluminate hydrate is observed, which subsequently, with an increase in the calcium content, gradually transforms into the more stable C_3_AH_6_ phase.

Moreover, aluminum actively passes into the crystalline phases of ettringite and calcium hydroaluminate; hence, its content in the amorphous C–(A–)S–H phase decreases. It should be noted that the increased ettringite content observed in the **CAA** sample may act as a risk factor for sulfate corrosion.

**Figure 6:**
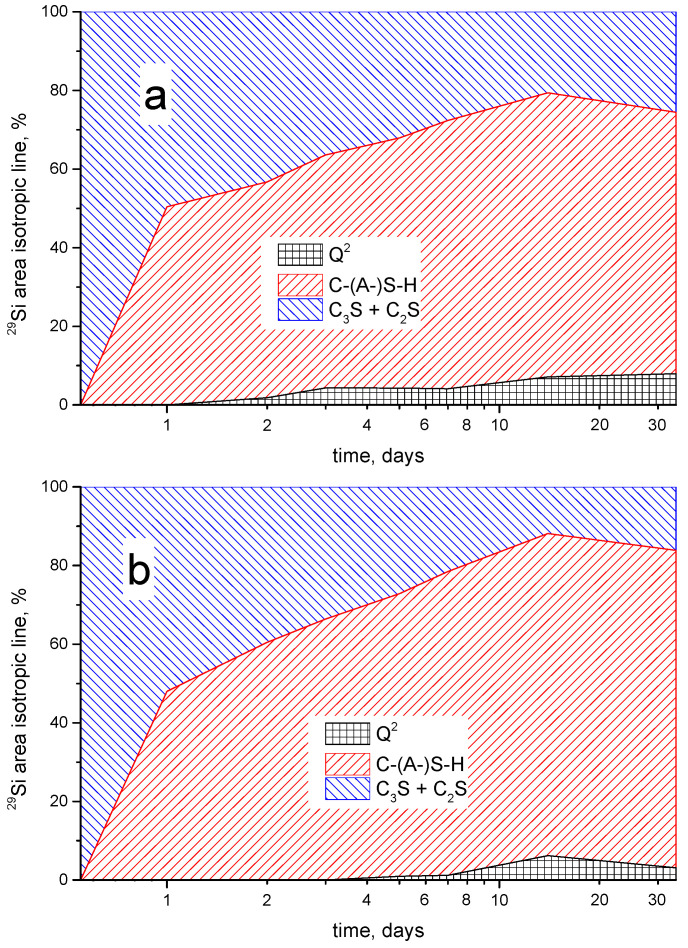

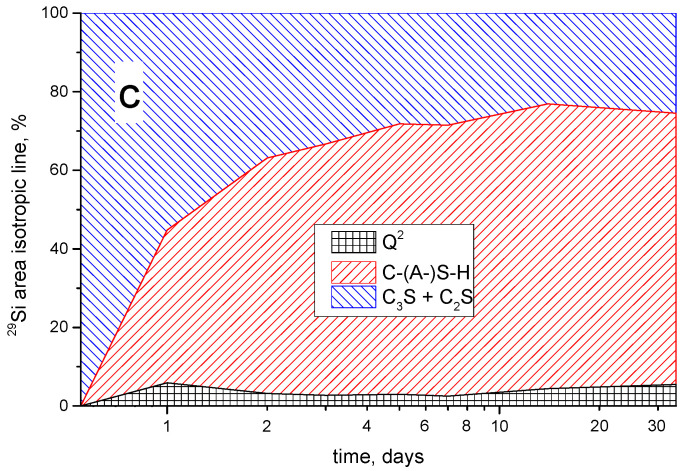
Time-evolution (in logarithmic scale) of the relative integrated areas of the isotropic components recognized in the ^29^Si NMR spectra of the samples: **C** (**a**), **CAA** (**b**) and **CSA** (**c**).

Figure 6 illustrates the time dependences of the relative integrated intensities of the ^29^Si NMR signals for all the three studied samples. When analyzing the change in the relative integrated intensities of the observed spectral components during hydration, we observed that the mass fraction of the silicate-containing clinker phases gradually decreases for all samples, while the mass fraction of the C–(A–)S–H phase increases proportionally, as well as the fraction of paired Q^2^ terahedra. It should be noted that the Q^2^ tetrahedra resolved in the spectrum appear on the first day for the **CSA** sample, on the second day for the **C** sample, and only on the third day for the **CAA** sample. Moreover, for the **CAA** sample, the spectral component, which is visually distinguishable from the baseline and is characteristic for Q^3^ and Q^4^ structural elements, also appears only on the second day of hydration.

These observations, along with the fact that no resolved peaks arise from other characteristic Q^1^, Q^2^ and Q^3^ structural elements [22], may indicate that such a characteristic layered structure of hydrated cements remains mainly amorphous; however, the number of paired Q^2^ tetrahedra increases, and this increase can correspond to an increase in the length of silicate chains, consisting of paired silicate tetrahedra. The presence of such phase corresponds to an increase in strength of the cement matrix. The formation of this phase for the **CAA** sample is observed at later stages of hydration.

**Figure 7:**
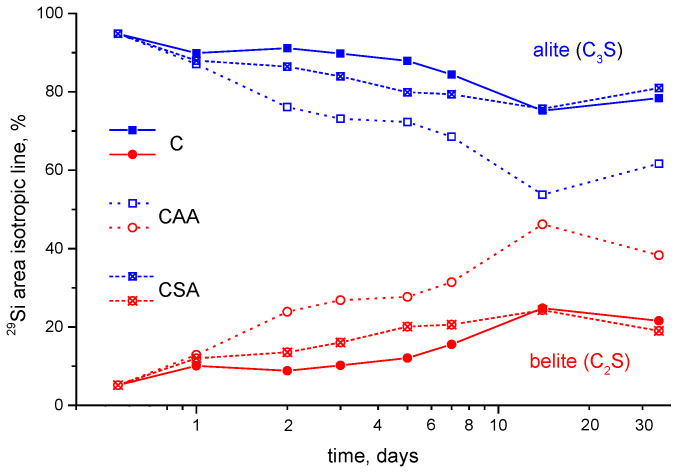
Time-evolution (in logarithmic scale) of the relative integrated areas of the isotropic component of calcium silicates (belite (C_2_S, blue markers) and alite (C_3_S, red markers)) recognized in the ^29^Si NMR spectra of the studied samples (**C** (filled points), **CAA** (empty points) and **CSA** (crossed points)).

It should be noted that, during cement hydration, a relative redistribution of the amounts of alite and belite occurs (Figure 7). For all the pastes, an increase in the relative content of belite is observed that is much larger for the **CAA** sample as compared to the other two.

Over the entire investigated time interval, the silicon-containing anhydrous phases did not completely hydrate. It should be noted that, in the **CAA** sample, the remaining amount of such phases is slightly less than for the other two samples; the addition of acetic acid leads to the involvement of a larger amount of alite in the formation of the C–(A–)S–H gel. That is, at the later stages of the hydration process of the **CAA** paste a smaller amount of alite remains anhydrated, and, thus, it is more effectively transformed to amorphous hydrate phase, affecting the strength properties of the hardened material.

## 5. Conclusions

In contrast to X-ray studies, one of the advantages of the NMR method is the ability to directly observe the signals of the nuclei both in the crystalline and amorphous local environments. As a result, in this work, it was possible to trace the time dependences of a set of chemical phases in the studied cement pastes. Despite the natural difficulty in obtaining unambiguous deconvolution of strongly overlapped signals of some cases, it was possible to identify the main components the presence of which is assumed in the chemistry of cementitious materials, including the amorphous phases, especially the crucial ones containing ^29^Si nuclei.

Considering all the above, it can be deduced that the addition of acetic acid and sodium acetate changes the kinetics of the cement paste phase composition during the hydration process. Adsorption of the acetate ion on the surface of the anhydrated and hydrated phases has a significant effect on the hydration process when the studied organic substances are added in the mixtures. Moreover, the presence of sodium ions slightly increases the alkalinity of the pore solution, partially reducing the efficiency of such adsorption.

It can be concluded that the addition of 3% acetic acid or sodium acetate, by cement mass, to the cement paste hindered the initial stages of the hydration process. The addition of sodium acetate led to the formation of a large amount of poorly soluble forms of calcium carbonate and a significant increase in the amount of polymerized silicon-containing phases.

Concerning the sulfate degradation of the cement paste, we see that the addition of acetic acid led to the development of favorable conditions for the formation of ettringite; in contrast, the addition of sodium acetate slightly slowed down this process. Thus, in the future studies, it would be interesting to investigate whether sodium acetate is a useful additive for improving the durability of hardened cementitious materials against sulfate attack. As only cement pastes were investigated in this work, further applied studies in this field are needed.

## Figures and Tables

**Table 1:** Quantitative phase analysis of Portland-limestone cement derived from Rietveld refinements of X-ray powder diffraction data (Bragg R factor −5.2%).

Mineral Phase	Formula *	wt.%
Alite	3CaO∙SiO_2_ (C_3_S)	56.2
Belite	2CaO∙SiO_2_ (C_2_S)	4.7
Tricalcium aluminate	3CaO∙Al_2_O_3_ (C_3_A)	5.8
Brownmillerite	4CaO∙Al_2_O_3_∙Fe_2_O_3_ (C_4_AF)	10.6
Gypsum	CaO∙SO_3_∙2H_2_O (C$\bar{S}$H_2_)	1.2
Basanite	2CaO∙2SO_3_∙H_2_O (C_2_$\bar{S}$_2_H)	<1.0
Anhydrite	CaO∙SO_3_ (C$\bar{S}$)	2.8
Periclase	MgO (M)	1.6
Calcite	CaO∙CO_2_ (C$\bar{C}$)	9.3
Dolomite	$\mathrm{CaO}\cdot \mathrm{MgO}\cdot 2{\mathrm{CO}}_{2}\;(\mathrm{CM}\bar{C}$_2_)	7.2

* The formulas in brackets correspond to cement chemist notation.

## Data Availability

Data available upon request to the authors.

## Supplementary Materials

The following are available online at https://www.mdpi.com/article/10.3390/ma15062004/s1. Figure S1: ^13^C NMR spectra of the anhydrated Portland-limestone cement and cement paste samples without additives (**C**) (a) and the chemical shift of spectra components (b) at the different hydration times. Deconvolution of C07 spectra per three (c) and two (d) components. Figure S2: ^13^C NMR spectra of the anhydrated Portland-limestone cement and cement paste samples with acetic acid (**CAA**) (a) and the chemical shift of spectra components (b) at the different hydration times. Figure S3: ^13^C NMR spectra of the anhydrated Portland-limestone cement and cement paste samples with sodium acetate (**CSA**) (a) and the chemical shift of spectra components (b) at the different hydration time. Figure S4: ^27^Al NMR spectra of the anhydrated Portland-limestone cement and cement paste sample without additives (**C**) (a) and the chemical shift of spectra components (b) at the different hydration time. Figure S5: ^27^Al NMR spectra of the anhydrated Portland-limestone cement and cement paste samples with acetic acid (**CAA**) (a) and the chemical shift of spectra components (b) at the different hydration time. Figure S6: ^27^Al NMR spectra of the anhydrated Portland-limestone cement and cement paste samples with sodium acetate (**CSA**) (a) and the chemical shift of spectra components (b) at the different hydration time. Figure S7: ^29^Si NMR spectra of the anhydrated Portland-limestone cement and cement paste sample without additives (**C**). Figure S8: ^29^Si NMR spectra of the anhydrated Portland-limestone cement and cement paste samples with acetic acid (**CAA**). Figure S9: ^29^Si NMR spectra of the anhydrated Portland-limestone cement and cement paste samples with sodium acetate (**CSA**).
