# Supplementary material for: 13C, 27Al and 29Si NMR Investigation of the Hydration Kinetics of Portland-Limestone Cement Pastes Containing CH3-COO−-R+ (R=H or Na) Additives
Source: Materials (Basel). 2022 Mar 8;15(6):2004. doi: 10.3390/ma15062004 (PMC8955463; doi:10.3390/ma15062004)
Supplement: Supplementary file 1 [file materials-15-02004-s001.zip › materials-1535705-supplementary.pdf]

materials

Supplementary Materials

# $^{13}C$, $^{27}Al$ and $^{29}Si$ NMR Investigation of the Hydration Kinetics of Portland-Limestone Cement Pastes Containing $CH_3$-$COO^-$-$R^+$ (R=H or Na) Additives

**Anton Mazur [1,*], Peter Tolstoy [1] and Konstantinos Sotiriadis [2,3]**

1 Saint Petersburg State University, Universitetsky pr. 26, 198504 Saint Petersburg, Russia; peter.tolstoy@spbu.ru
2 Institute of Theoretical and Applied Mechanics of the Czech Academy of Sciences, Prosecka 809/76, 19000 Prague, Czech Republic; sotiriadis@itam.cas.cz
3 Department of Building Materials and Products, South Ural State University (National Research University), pr. Lenina 76, 454080 Chelyabinsk, Russia
* Correspondence: a.mazur@spbu.ru

**Citation:** Mazur, A.; Tolstoy, P.; Sotiriadis, K. $^{13}C$, $^{27}Al$ and $^{29}Si$ NMR Investigation of the Hydration Kinetics of Portland-Limestone Cement Pastes Containing $CH_3$-$COO^-$-$R^+$ (R=H or Na) Additives. *Materials* **2022**, *15*, 2004. https://doi.org/10.3390/ma15062004

Academic Editors: Carlos Morón Fernández, Daniel Ferrández Vega and Jeong Gook Jang

Received: 16 December 2021
Accepted: 11 February 2022
Published: 8 March 2022

**Publisher's Note:** MDPI stays neutral with regard to jurisdictional claims in published maps and institutional affiliations.

**Copyright:** © 2022 by the authors. Licensee MDPI, Basel, Switzerland. This article is an open access article distributed under the terms and conditions of the Creative Commons Attribution (CC BY) license (http://creativecommons.org/licenses/by/4.0/).

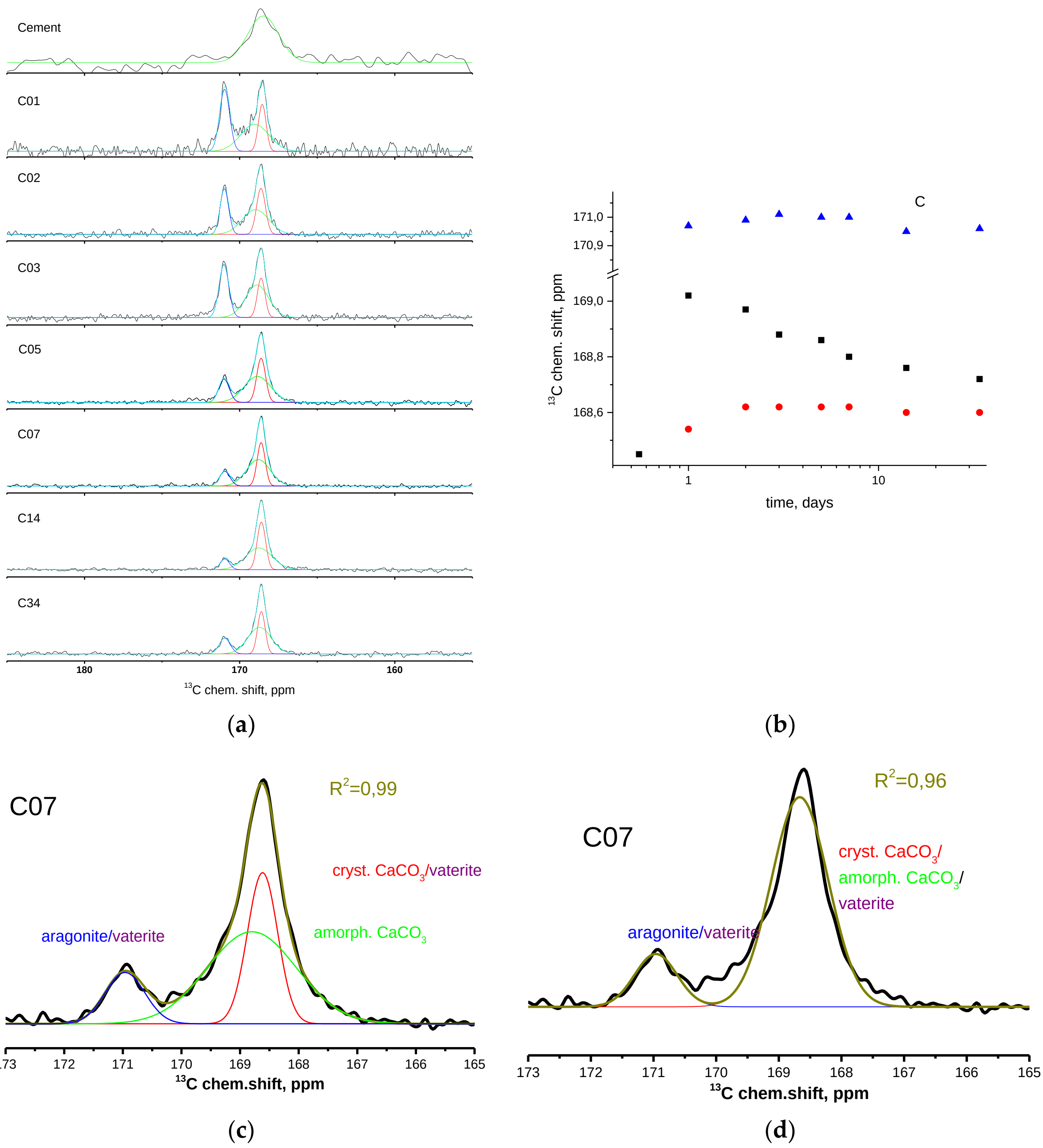


**Figure S1.** $^{13}C$ NMR spectra of the anhydrated Portland-limestone cement and cement paste samples without additives (**C**) (**a**) and the chemical shift of spectra components (**b**) at the different hydration time. Deconvolution of C07 spectra per three (**c**) and two (**d**) components.

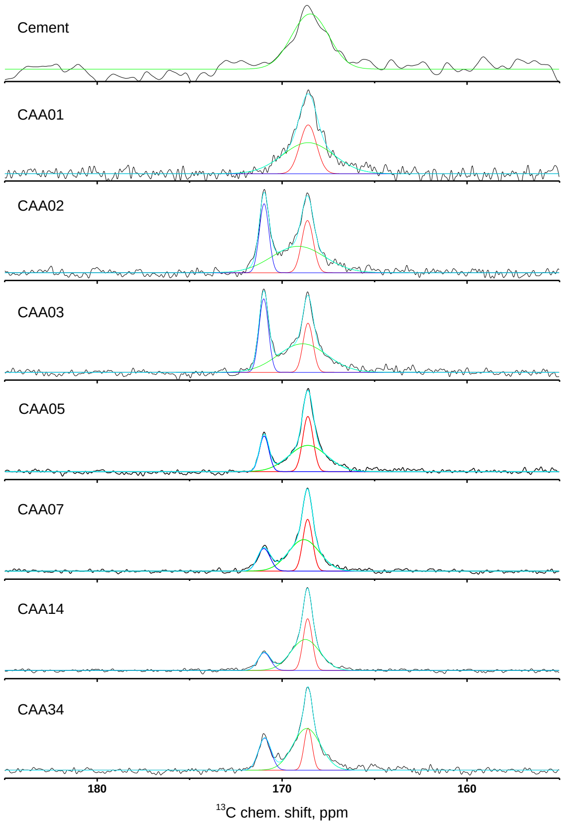


(**a**)

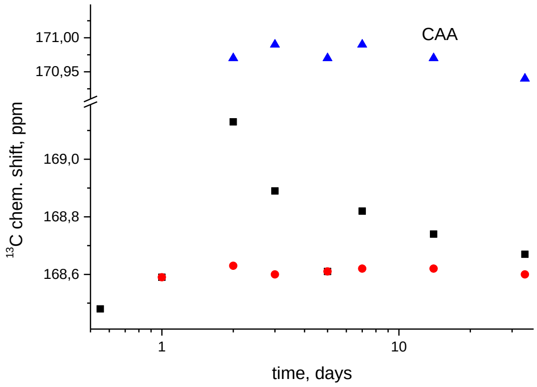


(**b**)

**Figure S2.** $^{13}C$ NMR spectra of the anhydrated Portland-limestone cement and cement paste samples with Acetic Acid (**CAA**) (**a**) and the chemical shift of spectra components (**b**) at the different hydration time.

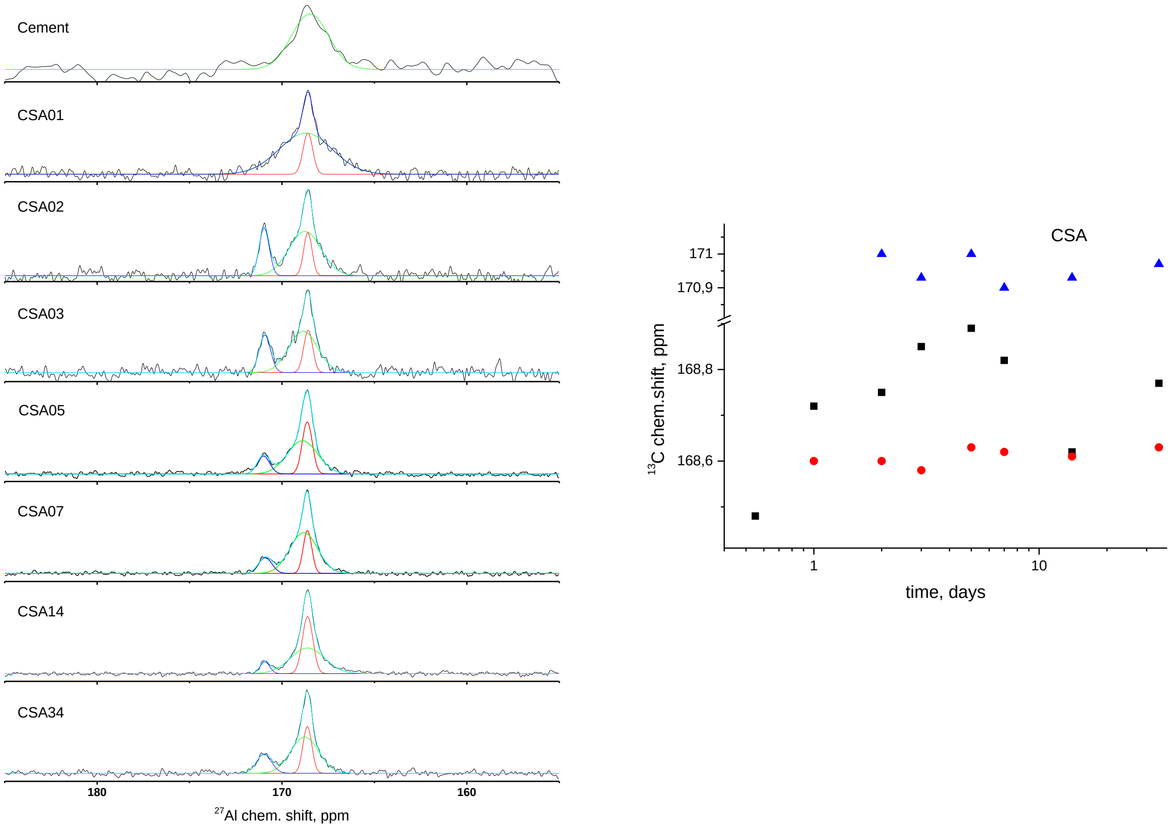


(**a**) (**b**)

**Figure S3.** $^{13}C$ NMR spectra of the anhydrated Portland-limestone cement and cement paste samples with Sodium Acetate (**CSA**) (**a**) and the chemical shift of spectra components (**b**) at the different hydration time.

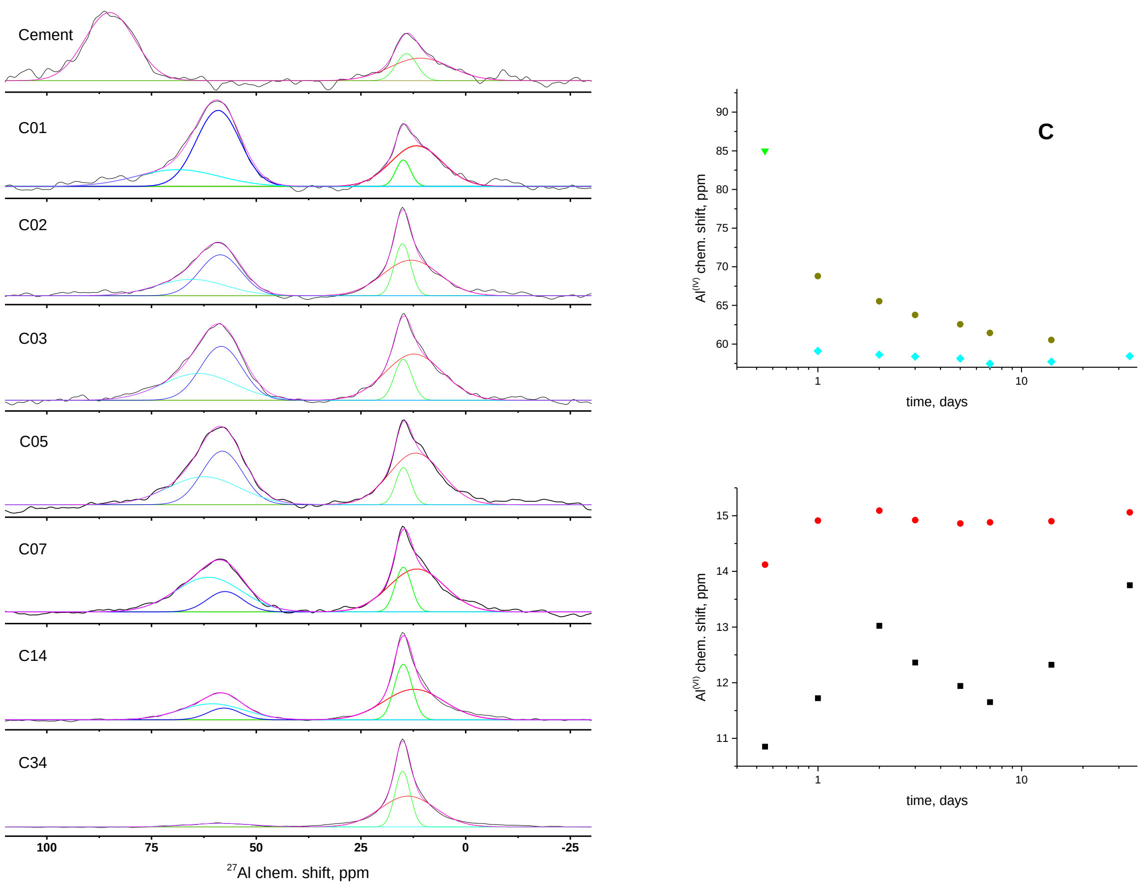


(**a**) (**b**)

**Figure S4.** $^{27}$Al NMR spectra of the anhydrated Portland-limestone cement and cement paste sample without additives (**C**) (**a**) and the chemical shift of spectra components (**b**) at the different hydration time.

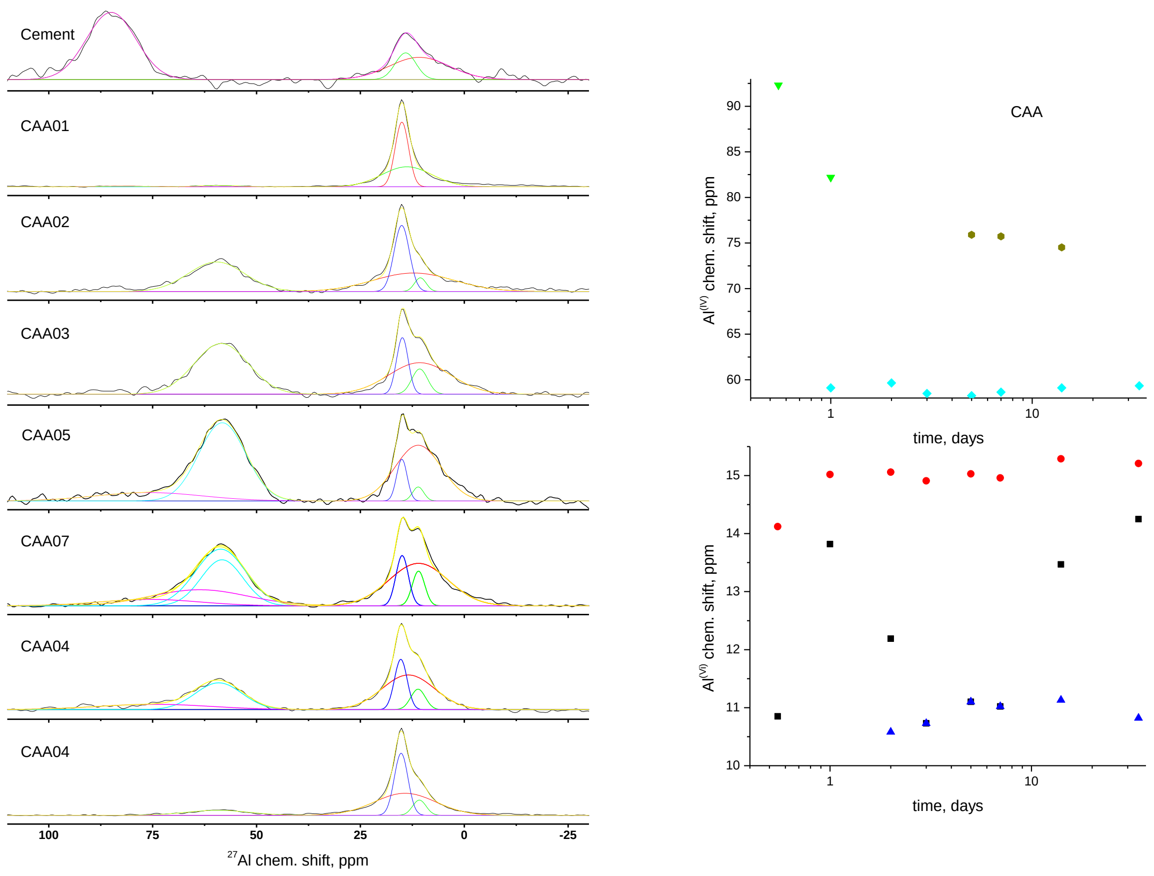


(**a**) (**b**)

**Figure S5.** $^{27}$Al NMR spectra of the anhydrated Portland-limestone cement and cement paste samples with Acetic Acid (**CAA**) (**a**) and the chemical shift of spectra components (**b**) at the different hydration time.

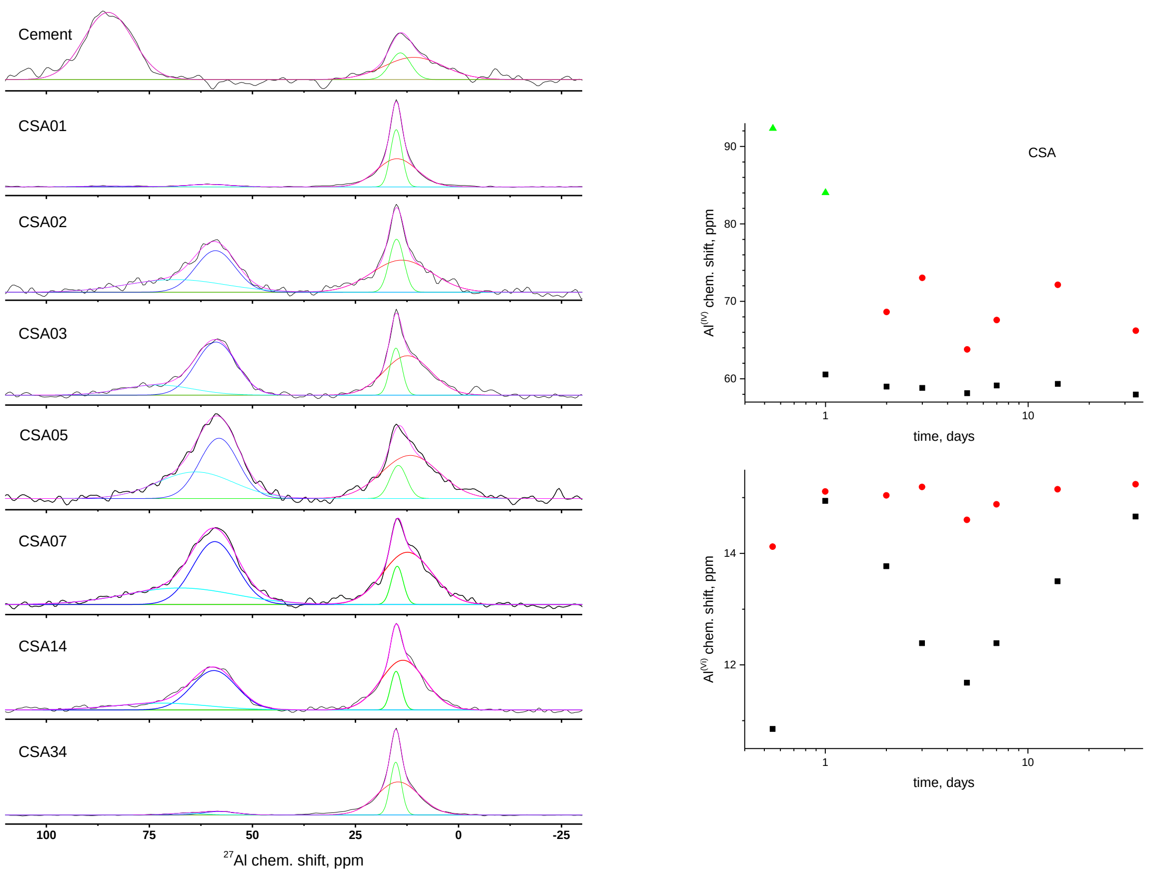


(**a**) (**b**)

**Figure S6.** $^{27}$Al NMR spectra of the anhydrated Portland-limestone cement and cement paste samples with Sodium Acetate (**CSA**) (**a**) and the chemical shift of spectra components (**b**) at the different hydration time.

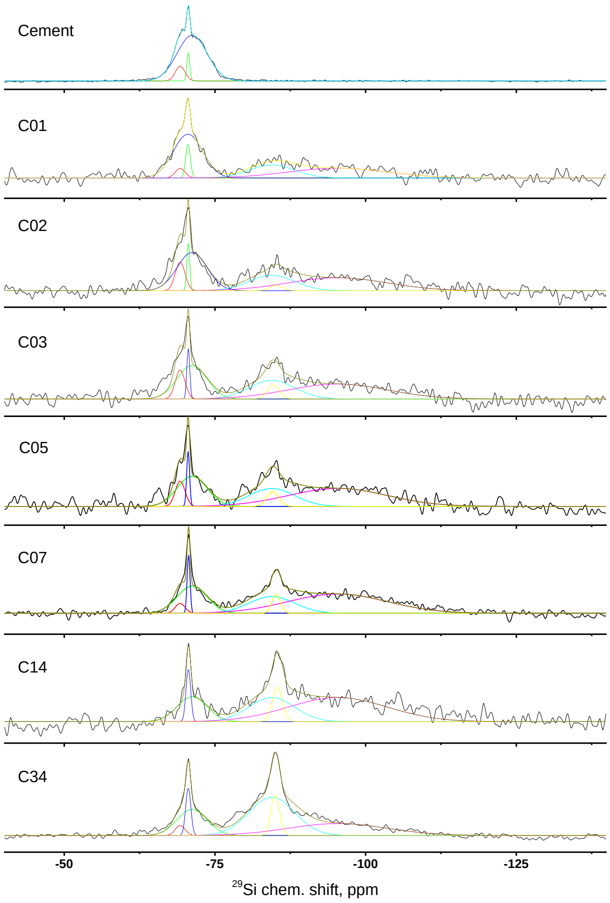


**Figure S7.** $^{29}$Si NMR spectra of the anhydrated Portland-limestone cement and cement paste sample without additives (**C**).

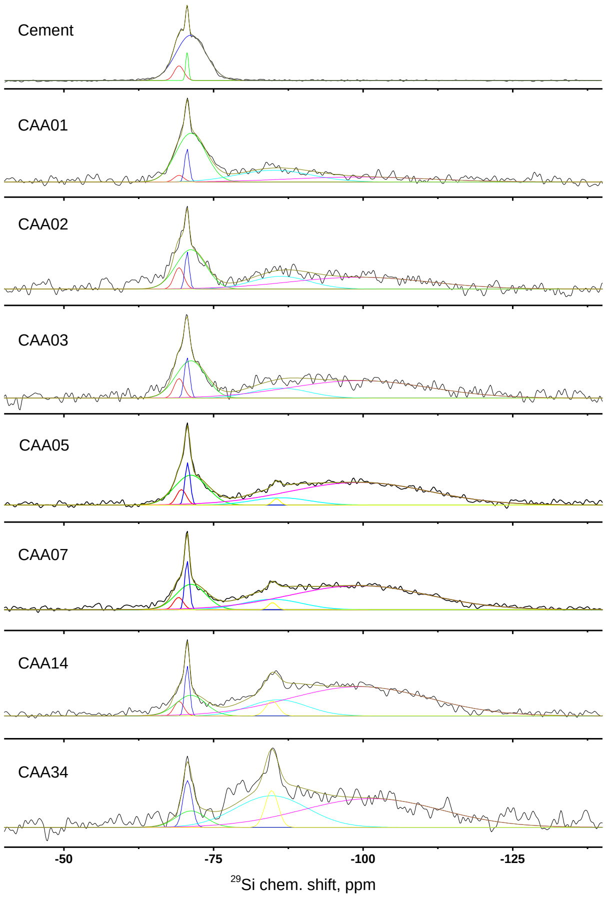


**Figure S8.** $^{29}Si$ NMR spectra of the anhydrated Portland-limestone cement and cement paste samples with Acetic Acid (**CAA**).

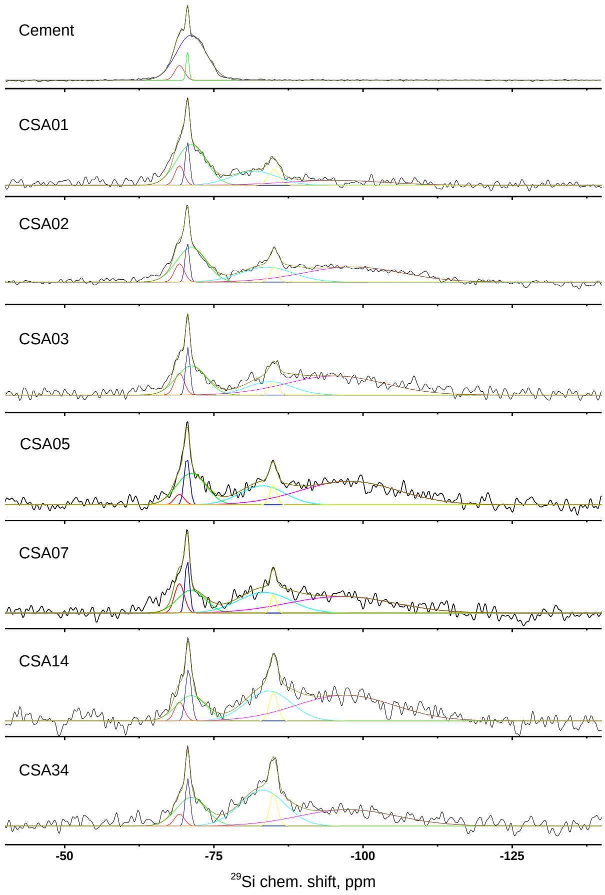


**Figure S9.** $^{29}$Si NMR spectra of the anhydrated Portland-limestone cement and cement paste samples with Sodium Acetate (**CSA**).
